# MRI-Targeted Prostate Fusion Biopsy: What Are We Missing outside the Target? Implications for Treatment Planning

**DOI:** 10.3390/curroncol31070308

**Published:** 2024-07-22

**Authors:** Marco Oderda, Alessandro Dematteis, Giorgio Calleris, Romain Diamand, Marco Gatti, Giancarlo Marra, Gilles Adans-Dester, Yazan Al Salhi, Antonio Pastore, Riccardo Faletti, Paolo Gontero

**Affiliations:** 1 Division of Urology, Department of Surgical Sciences, Molinette Hospital, University of Turin, 10126 Torino, Italy; dmtlsn@gmail.com (A.D.); giorgio.calleris@unito.it (G.C.); giancarlo.marra@unito.it (G.M.); paolo.gontero@unito.it (P.G.); 2 Department of Urology, Jules Bordet Institute—Erasme Hospital, Hôpital Universitaire de Bruxelles, Université Libre de Bruxelles, 1070 Brussels, Belgium; romain.diamand@ulb.be; 3 Division of Radiology, Department of Surgical Sciences, Molinette Hospital, University of Turin, 10126 Torino, Italy; marco.gatti@unito.it (M.G.); riccardo.faletti@unito.it (R.F.); 4 Department of Urology, Centre Hospitalier Universitaire Namur-Godinne, UCLouvain, 5530 Yvoir, Belgium; gilles.adans-dester@chuuclnamur.uclouvain.be; 5 Urology Unit, Department of Medico-Surgical Sciences and Biotechnologies, Sapienza University of Rome, 04100 Latina, Italy; yazan.alsalhi@uniroma1.it (Y.A.S.); antonioluigi.pastore@uniroma1.it (A.P.)

**Keywords:** prostate biopsy, fusion, out-field, outside, MRI, accuracy

## Abstract

**Introduction**: This study aimed to evaluate the added diagnostic value of systematic biopsies (SBx) after magnetic resonance imaging (MRI)-targeted biopsies (TBx) and the presence of prostate cancer (PCa) outside MRI targets, in a prospective, contemporary, multicentric series of fusion biopsy patients. **Methods**: We collected data on 962 consecutive patients who underwent fusion biopsy between 2022 and 2024. Prostate cancer was considered clinically significant (csPCa) in the case of grade ≥ 2. Median test and Fisher exact chi-square tests were used. To identify predictors of out-field positivity, univariate and multivariable logistic regression analyses were performed. **Results**: Prostate cancer and csPCa were detected by TBx only in 56% and 50%, respectively, and by SBx only in 55% and 45%, respectively (*p* < 0.001). Prostate cancer and csPCa were diagnosed by TBx in 100 (10%) and 82 (8%) SBx-negative cases and by SBx in 86 (9%) and 54 (6%) TBx-negative cases (*p* < 0.001). Tumors outside MRI targets were found in 213 (33%) cases in the same lobe and 208 (32%) in the contralateral lobe, most of them being csPCa. Predictors of out-field contralateral PCa were positive DRE (HR 1.50, *p* 0.03), PSA density ≥ 0.15 (HR 2.20, *p* < 0.001), and PI-RADS score 5 (HR 2.04, *p* 0.01). **Conclusions**: Both TBx and SBx identify a non-negligible proportion of csPCa when the other modality is negative. SBx after TBx should always be considered given the risk of missing other csPCa foci within the prostate, especially in patients with positive DRE, PSA density ≥ 0.15, and PIRADS 5 lesions.

## 1. Introduction

Multiparametric magnetic resonance imaging (MRI) is essential in the diagnosis of prostate cancer (PCa) and is recommended in patients with clinical suspicion of PCa. If a suspicious lesion is found on MRI, MRI-targeted prostate biopsy (TBx) is advised, together with a systematic mapping [1]. The need for systematic biopsies (SBxs) is supported by a risk of missing approximately 16% and 10% of clinically significant PCa (csPCa) in biopsy-naïve and repeat-biopsy patients, according to a recent Cochrane meta-analysis [2]. However, the performance of supplementary mapping is increasingly being questioned, considering that several studies demonstrated a limited added diagnostic benefit of 5% to 7% [3,4]. A recent multicentric study that evaluated the added value in csPCa detection of side-specific SBx relative to an MRI lesion found that biopsies taken at the opposite side of the MRI-suspicious lesion have only a negligible impact on per-patient cancer detection [5]. Nevertheless, SBx has been suggested to detect a high number of PCa foci outside mpMRI targets, improving the assessment of the tumor burden inside the prostate [6]. Considering the multifocal nature of PCa, a thorough knowledge of the location of all cancer foci inside the prostate is essential for a correct treatment decision. In the present study, we evaluated the added diagnostic value of SBx and the presence of PCa outside MRI targets, in a prospective, contemporary, multicentric series of fusion biopsy patients.

## 2. Patients and Methods

### 2.1. Study Population

After obtaining institutional review boards’ approvals, data from 962 consecutive patients who underwent TBx and SBx between April 2022 and January 2024 were prospectively collected at three European referral centers. All patients signed informed consent for the use of clinical information for clinical studies (coordinator ethics committee protocol number: 0040478). This study was performed according to the Standard for Reporting Diagnostic Accuracy Studies [7].

### 2.2. MRI and Biopsy Techniques

All prebiopsy MRIs were performed using a 1.5-T or 3-T scanner with a surface coil and consisted of multiplane T1- and T2-weighted imaging, diffusion-weighted imaging (DWI), and dynamic contrast enhancement. Scans were reviewed and scored by experienced radiologists using the PI-RADS v.2 or 2.1 protocols. All MRIs had at least one lesion suspicious for PCa, defined as PI-RADS score ≥ 3. All MRI-targeted prostate biopsies were performed with a transperineal approach under local anesthesia with the Koelis Trinity^®^ (Koelis, Meylan, France) (*n* = 746) or Esaote MyLab™ X9 (Esaote, Genova, Italy) (*n* = 216) fusion imaging system. Expert operators (experience > 500 fusion biopsies) performed or oversaw all the biopsies. A minimum of 2 targeted cores per target were taken, followed by systematic biopsies. SBx adopted a standardized template to sample the posterior region of the prostate, typically with 6 cores per lobe, irrespective of the location of the MRI target.

### 2.3. Endpoints and Statistical Analyses

The primary endpoint of this study was the added diagnostic value of each biopsy modality, with a side-specific evaluation. For this aim, a per-patient analysis was conducted, considering the lesion with a higher PI-RADS score in the case that multiple targets were reported at MRI. The secondary endpoint was the rate of cancer positivity outside MRI targets at SBx, within the same lobe or contralateral. For this purpose, a post hoc analysis was performed on the 643 patients with a positive biopsy, analyzing the location of MRI targets and the location of positive SBx cores. PCa was considered clinically significant (csPCa) in the case of ISUP grade ≥ 2. Continuous data were reported as medians and interquartile range (IQR) and categorical parameters were shown as counts and percentages. The median test and Fisher exact chi-square test were used when appropriate to compare continuous and categorical variables. To identify predictors of out-field positivity, univariate logistic regression was performed initially to obtain unadjusted odds ratios. Subsequently, all variables were put into a multivariable model to obtain adjusted hazard ratios. Variables of interest for logistic regression were PSA, digital rectal examination (DRE), PSA density (as a binary variable, adopting a cut-off of 0.15), previous negative biopsy status, PI-RADS score, and diameter of MRI target. Statistical significance was set at two-sided *p* < 0.05. Statistical analyses were performed with SPSS version 29.0 (IBM Corp, Armonk, NY, USA).

## 3. Results

Baseline patient characteristics are shown in Table 1. The patients’ median age was 71 (IQR 65–77) and median PSA was 7.0 ng/mL (IQR 5.1–10.2), with a median PSA density of 0.15 (0.09–0.23). Most patients were biopsy-naïve (82%) and had a single MRI target (78%). Targets were scored as 3, 4, and 5 in 21%, 57%, and 22%, respectively, and were more often situated in the posterior (89%) and equatorial (43%) regions. The median number of cores taken was 3 (IQR 3–3) per target and 12 (IQR 12–12) during SBx.

### 3.1. Added Diagnostic Value of TBx and SBx

The biopsy results are shown in Table 2. The cancer detection rate of TBx + SBx was 65% for all cancers and 55% for csPCa. Taken individually, TBx diagnosed PCa and csPCa in 56% and 50%, respectively, whereas SBx was slightly inferior, with a detection rate of 55% and 45%, respectively (*p* < 0.001). Grades 2 (27%) and 3 (19%) were the most frequent diagnoses. There was a statistically significant difference for csPCa detection in favor of TBx over SBx (*p* < 0.001). TBx was able to detect PCa and csPCa in 100 (10%) and 82 (8%) cases where SBx was negative for PCa and csPCa, respectively (*p* < 0.001). On the other hand, SBx detected PCa and csPCa in 86 (9%) and 54 (6%) cases where TBx was negative for PCa and csPCa, respectively (*p* < 0.001). When TBx was positive, SBx led to an upgrade in the final ISUP score in 46 (5%) cases.

### 3.2. Cancer Positivity Outside MRI Targets at SBx

**Figure 1:**
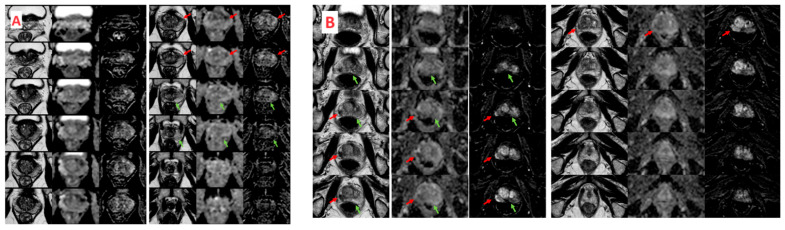
(**A**): *Out-field positivity in the same lobe of the MRI target.* MRI detected a PIRADS 4 lesion (red arrow) in the left anterior peripheral zone at the mid-portion of the prostate. This finding was confirmed by fusion biopsy, which identified ISUP 3 adenocarcinoma in the target area. Additionally, a focus of ISUP 3 adenocarcinoma was found in the left paramedian apical region where the MRI was negative (green arrow). (**B**): *Out-field positivity in the lobe contralateral to the MRI target.* MRI detected a PIRADS 5 lesion in the right peripheral zone in the posterolateral sector of the basal and middle zone (red arrow). Fusion biopsy identified ISUP 5 adenocarcinoma in the target area. Additionally, ISUP 5 adenocarcinoma was also found in the contralateral lobe, in the left peripheral zone at the base in the posteromedial sector, where the MRI (green arrow) showed no significant changes.

Among the 625 positive biopsies on TBx + SBx, out-field positivity (outside MRI targets) was found in 213 (33%) cases in the same lobe of the MRI target, and 208 (32%) cases in the contralateral lobe (Figure 1). Focusing only on csPCa, 195 (30%) were diagnosed in the same lobe but outside MRI targets, and 176 (27%) in the contralateral lobe. The results of cancer positivity outside MRI targets with grade distribution are shown in Table 3, together with the regression analyses. Most missed cancers were ISUP 2 (14% and 13% in the ipsilateral and contralateral lobes, respectively). Around 5% of missed cancers were represented by ISUP 4 and 5. Based on the multivariable analysis, predictors of out-field positivity in the same lobe of the target were positive DRE (HR 1.76, 95%CI 1.23–2.51, *p* 0.002), PSA density ≥ 0.15 (HR 1.61, 95%CI 1.21–3.85, *p* 0.007), and PI-RADS score 5 (HR 2.16, 95%CI 1.21–3.85, *p* 0.009). The same variables were also predictors of contralateral out-field positivity as follows: positive DRE (HR 1.50, 95%CI 1.03–2.17, *p* 0.03), PSA density ≥ 0.15 (HR 2.20, 95%CI 1.53–3.16, *p* < 0.001), and PI-RADS score 5 (HR 2.04, 95%CI 1.15–3.61, *p* 0.01).

## 4. Discussion

The role achieved by MRI in the diagnostic pathway of PCa is not in question and is supported by strong evidence [1,8]. The PRECISION study demonstrated that MRI-targeted biopsy in men with an MRI-suspicious lesion leads to more significant cancers being identified and fewer insignificant cancers being diagnosed. The European guidelines recommend the execution of systematic mapping after TBx, given the potential presence of mpMRI-invisible csPCa, estimated at 10–20% [9]. However, the diagnostic benefit of SBx has been recently reduced to 5–7% [3,4,10]. In a recent multicentric study, we showed that SBx in addition to TBx improved the detection rate only by 4% for all PCa and by 3% for csPCa [6]. Similar results were found by Cauli et al., who showed an increase of 3% for all PCa and csPCa [11]. Detractors of SBx claim that the performance of SBx is typically overestimated because of test review bias, given that the physician performing systematic mapping is aware of the location of the MRI lesion and somehow targets the lesion even during systematic mapping [12].

The present study is aligned with the recent literature [3,5,6,13] concerning the superiority of TBx over SBx in terms of the detection rate of all PCa and csPCa. Nevertheless, our results showed that both TBx and SBx identify a non-negligible proportion of csPCa when the other modality is negative (10% for TBx, 9% for SBx), in line with the PAIREDCAP study that concluded that combining targeted and systematic biopsy offers the best chances of detecting the cancer [14].

More importantly, in our study, we focused on the rate of cancer positivity found outside MRI targets, even when the MRI target correctly led to a cancer diagnosis. This issue is essential for treatment decision planning, as the presence of csPCa in other regions of the prostate beyond the MRI-visible lesion could lead to abandoning active surveillance or focal treatment or could make it harder to propose a full nerve-sparing surgery, depending on the burden of csPCa. In a series of 1.992 fusion biopsy patients, we previously demonstrated an alarming rate of 57% out-field positivity, of which 58% were clinically significant, even though that study was limited by the lack of data on the precise location of systematic cores [6]. The present study showed that 30% of our patients with a positive biopsy had at least one core of csPCa in the same lobe but outside the MRI target, and—even more alarmingly—27% harbored at least one core of csPCa in the lobe contralateral to the MRI target. Previously, Choi et al. evaluated 185 candidates for hemiablation and found that 123 (66%) of them had bilateral cancer after radical prostatectomy and 73 (39%) had csPCa in MRI-negative lobes [15]. More recently, Gunzel et al. found that 145 out of 736 (20%) patients with unilateral suspicious lesions on MRI were detected with contralateral PCa-positive SBx. Overall, 238 of their patients (25%) showed positive SBx outside of the described PI-RADS lesions [16]. Fletcher et al. evaluated a series of 346 patients with a pre-biopsy MRI and a PI-RADS ≥ 3 lesion. When TBx was positive, detection of higher grade csPCa on SBx compared with TBx occurred in only 5% of cases [17]. A retrospective analysis of 2.048 fusion biopsy patients by Brisbane et al. found that 90% of csPCa cores detected by SBx were confined in the so-called “penumbra”, within a radius of 10 mm from the nearest lesion. Nevertheless, in 18% of patients, csPCa was diagnosed only by sampling outside the MRI lesion, with the yield decreasing with increasing distance [18]. Bonekamp et al. confirmed these findings, with 18% of csPCa discovered outside mpMRI regions even when a 10 mm security margin was adopted, indicating that prostate MRI has limited ability to completely map all cancer foci within the prostate [19]. Our rates of out-field csPCa positivity are even higher, indicating a contralateral disease in 32% of cases. To better identify patients at risk of cancer outside MRI targets, positive DRE, PSA density ≥ 0.15, and PI-RADS 5 score can be used as prognostic variables. PSA density was previously found to be associated with the presence of MRI-negative PCa, together with the black race [20]. Recently, Noujeim et al. evaluated the distance of positive SBx from the index lesion and developed a three-tier prediction model, where the only predictive factors for positive SBx were the PI-RADS score and PSA density [21].

All things considered, the omittance of SBx after MRI-targeted biopsies should be discouraged at present given the risk of missing other csPCa foci within the prostate, which might jeopardize subsequent cancer management. Among the strengths of our study is the prospective and multicentric design. The limitations of our study are the presence of multiple operators, different settings and types of MRIs, different habits in performing SBx, and the absence of central radiological and pathological revision. MRIs were performed in both high- and low-volume centers, and **their** quality was not centrally reviewed, which might have introduced bias. On the other hand, biopsies were performed in referral centers with a high level of expertise.

## 5. Conclusions

Both TBx and SBx identify a non-negligible proportion of csPCa when the other modality is negative. The performance of SBx after TBx should always be considered given the risk of missing other csPCa foci within the prostate, especially in patients with positive DRE, PSA density ≥ 0.15, and PIRADS 5 lesions.

## Figures and Tables

**Table 1:** Descriptive characteristics.

Variable	Total (n = 962)
Age at biopsy, yr, median (IQR)	71 (65–77)
PSA, ng/mL, median (IQR)	7.0 (5.1–10.2)
PSA density, median (IQR)	0.15 (0.09–0.23)
Suspicious DRE, *n* (%)	281 (29.2)
Biopsy status	
- Naïve.	794 (82.5)
- Previous negative biopsy.	168 (17.5)
Prostate volume at mpMRI, cc, median (IQR)	48.9 (35.0–65.0)
Number of suspicious lesions, *n* (%)	
- Single.	750 (78.0)
- Multiple.	212 (22.0)
PI-RADS score of index lesion, n (%)	
- 3.	202 (20.9)
- 4.	548 (56.9)
- 5.	212 (22.2)
Maximum target diameter of index lesion, mm, median (IQR)	10.0 (8.0–15.0)
ADC value of index lesion, median (IQR)	0.7 (0.6–0.8)
Target(s) location, *n* (%) *	
- Anterior.	176 (18.3)
- Posterior.	615 (88.9)
- Transitional.	134 (13.9)
Target(s) location, *n* (%) *	
- Apex.	340 (35.3)
- Equator.	412 (42.8)
- Base.	229 (23.8)
*N* of cores taken per target at targeted biopsy, median (IQR)	3 (3–3)
*N* of cores taken at systematic biopsy, median (IQR)	12 (12–12)

Legend: ADC = apparent diffusion coefficient; DRE = digital rectal examination; IQR = interquartile range; mpMRI = multiparametric magnetic resonance imaging; PI-RADS = Prostate Imaging Reporting & Data System * multiple locations allowed per target.

**Table 2:** Cancer detection rates of targeted and systematic biopsies.

Variable (Cohort N 980)	Targeted Biopsy	Systematic Biopsy	Targeted + Systematic Biopsy
Number of positive cores, median (IQR)	2 (0–3)	2 (0–4)	-
Biopsy positive for PCa, *n* (%)	539 (56.0)	525 (54.6)	625 (64.9)
Biopsy positive for csPCa, *n* (%)	486 (50.5)	433 (45.0)	555 (57.7)
Biopsy highest ISUP grade, *n* (%)			
- 0.	86 (8.9)	100 (10.4)	-
- 1.	53 (5.5)	92 (9.5)	70 (7.3)
- 2.	232 (24.1)	220 (22.8)	261 (27.1)
- 3.	166 (17.2)	133 (13.8)	184 (19.1)
- 4.	57 (5.9)	51 (5.3)	73 (7.6)
- 5.	31 (3.2)	29 (3.0)	37 (3.8)
Biopsy positive for PCa when the other modality is negative, *n* (%)	100 (10.4)	86 (8.9)	-
Biopsy positive for csPCa when the other modality is negative, *n* (%)	82 (8.5)	54 (5.6)	-

Legend: IQR = interquartile range; mpMRI; ISUP = International Society of Urological Pathology; PCa = prostate cancer; csPCa = clinically significant prostate cancers.

**Table 3:** Cancer positivity outside MRI targets at systematic mapping.

Variable (Cohort *N* 643)		All PCa	csPCa Only
Out-field positivity of SBx in the same lobe, *n* (%)		213 (33.1)	195 (30.3)
- ISUP grade 1.		39 (6.0)	-
- ISUP grade 2.		93 (14.4)	93 (14.4)
- ISUP grade 3.		47 (7.3)	47 (7.3)
- ISUP grade 4.		26 (4.0)	26 (4.0)
- ISUP grade 5.		8 (1.2)	8 (1.2)
- With MRI target(s) positive, *n* (%).		194 (30.2)	162 (25.2)
- With MRI target(s) negative, *n* (%).		19 (2.9)	12 (1.8)
Out-field positivity of SBx in the contralateral lobe, *n* (%)		208 (32.3)	176 (27.4)
- ISUP grade 1.		32 (4.9)	-
- ISUP grade 2.		82 (12.7)	82 (12.7)
- ISUP grade 3.		48 (7.4)	48 (7.4)
- ISUP grade 4.		30 (4.6)	30 (4.6)
- ISUP grade 5.		16 (2.5)	16 (2.5)
- With MRI target(s) positive, *n* (%).		179 (27.8)	158 (24.6)
- With MRI target(s) negative, *n* (%).		29 (4.5)	18 (2.8)
**Predictors of out-field positivity in the same lobe**			
	**Univariate analysis**	**Multivariate analysis**	
PSA (ng/mL)	1.00 (0.99–1.00), *p* 0.66	-	
Positive DRE	2.03 (1.48–2.80), *p* < 0.001	1.76 (1.23–2.51), *p* 0.002	
PSA density ≥ 0.15	1.76 (1.28–2.43), *p* < 0.001	1.61 (1.21–3.85), *p* 0.007	
Previous negative biopsy	0.73 (0.48–1.12), *p* 0.15	-	
PI-RADS score			
- 3.	Ref	Ref	
- 4.	1.40 (0.90–2.17), *p* 0.12	1.50 (0.93–2.40), *p* 0.09	
- 5.	2.61 (1.61–4.24), *p* < 0.001	2.16 (1.21–3.85), *p* 0.009	
Lesion diameter, mm	1.03 (1.00–1.05), *p* 0.005	1.00 (0.97–1.03), *p* 0.94	
**Predictors of out-field positivity in the contralateral lobe**			
	**Univariate analysis**	**Multivariate analysis**	
PSA (ng/mL)	1.00 (1.00–1.00), *p* 0.33	-	
Positive DRE	1.90 (1.37–2.63), *p* < 0.001	1.50 (1.03–2.17), *p* 0.03	
PSA density ≥ 0.15	2.19 (1.57–3.06), *p* < 0.001	2.20 (1.53–3.16), *p* < 0.001	
Previous negative biopsy	0.67 (0.43–1.04), *p* 0.07	-	
PI-RADS score			
- 3.	Ref	Ref	
- 4.	0.99 (0.65–1.51), *p* 0.97	0.96 (0.60–1.55), *p* 0.89	
- 5.	2.43 (1.53–3.86), *p* < 0.001	2.04 (1.15–3.61), *p* 0.01	
Lesion diameter, mm	1.03 (1.01–1.05), *p* 0.003	1.00 (0.97–1.03), *p* 0.80	

Legend: DRE = digital rectal examination; IQR = interquartile range; mpMRI; ISUP = International Society of Urological Pathology; PCa = prostate cancer; csPCa = clinically significant prostate cancer; SBx = systematic biopsy.

## Data Availability

The data presented in this study are available on request from the corresponding author.
